# Spatially Resolved Plant Metabolomics

**DOI:** 10.3390/metabo15080539

**Published:** 2025-08-08

**Authors:** Ronald J. Myers, Zachary M. Tretter, Abigail G. Daffron, Eric X. Fritschi, William Thives Santos, Maiya L. Foster, Matthew Klotz, Kristin M. Stafford, Christina Kasch, Thomas J. Taylor, Lillian C. Tellefson, Tyler Hartman, Dru Hackler, Preston Stephen, Lloyd W. Sumner

**Affiliations:** Division of Biochemistry, University of Missouri-Columbia, Columbia, MO 65211, USA; rjm4kd@missouri.edu (R.J.M.J.); ztmnb@missouri.edu (Z.M.T.); agdmf8@missouri.edu (A.G.D.); efgcc@missouri.edu (E.X.F.); gthivessantos@missouri.edu (W.T.S.); mlfcpy@missouri.edu (M.L.F.); mrkx86@missouri.edu (M.K.); staffordk@missouri.edu (K.M.S.); cmkg4y@missouri.edu (C.K.); tjt759@missouri.edu (T.J.T.); lcthg4@health.missouri.edu (L.C.T.); tjh8d3@missouri.edu (T.H.); ddhfwt@missouri.edu (D.H.); pdshmc@missouri.edu (P.S.)

**Keywords:** spatial metabolomics, mass spectrometry, desorption electrospray ionization, matrix-assisted laser desorption ionization, plant metabolism, plant stress, mass spectral imaging

## Abstract

Research and innovation in metabolomics tools to measure metabolite accumulation within plants have led to important discoveries with respect to the improvement of plant stress tolerance, development, and crop yield. Traditional metabolomics analyses have commonly utilized gas chromatography–mass spectrometry and liquid chromatography–mass spectrometry, but these methods are often performed without regard for the spatial locations of metabolites within tissues. Methods for mass spectral imaging (MSI) have recently been developed to detect and spatially resolve metabolite accumulation and are rapidly being adopted on a wider scale. Since 2010, the number of publications incorporating mass spectral imaging has grown from approximately 80 articles to over 378 on a yearly basis, constituting an increase of at least 350% during this time frame. Spatially resolved metabolite accumulation data provides unique insights into the function and regulation of plant biochemical pathways. Mass spectral imaging is commonly paired with desorption ionization technologies, including matrix-assisted laser desorption ionization (MALDI) and desorption electrospray ionization (DESI), to generate accurate, spatially resolved metabolomics data from prepared tissue segments. Here, we describe the most recent advancements in sample preparation methods, mass spectral imaging technologies, and data processing tools that have been developed to address the limits of MSI technology. Additionally, we summarize recent applications of MSI technologies in plant metabolomics and discuss potential avenues for future research advancements within the plant biology community through the use of these technologies.

## 1. Introduction

Metabolomics is a maturing science that is focused on the large-scale study of metabolism within an organism. Conceptually described and first termed in 1998, the metabolome is the ultimate product of the central dogma of molecular biology and is generally considered to encompass all small molecules within an organism (<1500 Daltons in size) that are involved in cellular metabolism and determination of the organismal phenotype [1,2,3]. Historically, plant scientists have played a major role in the development of the field [4,5]. With estimations of as many as 200,000–1,000,000 different metabolites being present across various plant species that function to give rise to unique phenotypic variations throughout the plant kingdom, metabolomics has been of particular interest to the field of plant science [6,7]. Recent achievements and the utility of plant metabolomics have been the subject of multiple reviews [8,9].

Many metabolomics approaches have been described and reviewed in the literature that analyzed tissue samples with limited regard to spatial considerations within the organism of interest [10]. Recently, technologies have emerged that enable spatial determination of metabolites within a tissue [11,12]. With plant metabolism being spatially organized and regulated within subcellular organelles, as well as tissues, organs, and even single-cell-specific metabolic regulation being present within plants, it is vital to expand upon our current knowledge of the metabolome with respect to the locations of metabolite regulation using the maximum achievable cellular resolution. In this review, we advocate for continued adoption of mass spectral imaging technologies within plant research workflows and review recent technological advances and applications in spatially resolved plant metabolomics.

### 1.1. Why Do We Need Spatial Metabolomics?

Spatially resolved metabolite localization and regulation enable a deeper understanding of metabolic function and metabolite-induced phenotypic modulation of complex, multicellular organisms. Although traditional metabolomics approaches have provided many critical biological insights that have led to improvements in plant yield, growth, stress resilience, and development, a significant amount of potentially transformative information has been lost due to knowledge gaps that occur as a result of traditional metabolite extraction processes from homogenized tissues and the subsequent inability to map the acquired information back to the spatial location of origin [9,13,14,15,16].

Utilization of spatial metabolomics technologies has recently been employed to begin addressing this important limitation within the metabolomics field. Mass spectral imaging (MSI) allows for resolving metabolite localization at tissue-specific, cell-specific, and subcellular levels in a spatial manner through coordinated x, y, and z instrumental raster scanning across a tissue of interest in conjunction with the acquisition of mass spectrometry data for each pixel within the scan. Recent advancements in applications of MSI have investigated traits such as plant stress tolerance [17]; these will be discussed in detail in Section 3. In addition to the discovery of novel roles of metabolites within plant physiological responses, spatial metabolomics can complement other multiomics technologies. Multiomics workflows that combine other omics technologies, such as genomics and transcriptomics, with MSI provide a powerful method for achieving deeper biological insights, including correlation of metabolic phenotypes with gene expression to support functional genomics approaches and pathway elucidation [18].

Spatial metabolomics techniques are commonly performed using ionization techniques such as matrix-assisted laser desorption ionization (MALDI) and desorption electrospray ionization (DESI). MSI technologies operate under either vacuum or ambient conditions and can generate spatial distribution data that provide a more complete image of the complex metabolic networks present in plants and allow for visual mapping of the metabolome within a tissue across space. Recent technological advancements in MALDI and DESI will be described later in Section 2.

Biological studies can now begin mapping metabolites back to their location within the analyzed tissue changes for a higher-resolution understanding of the plant’s metabolome during development or in response to stimuli, including factors such as abiotic stress, biotic stress, and cell-to-cell signaling responses. Ultimately, it may be possible to utilize metabolite mapping to selectively target specific cell types for metabolic adjustment using targeted genetic strategies and increase stress resiliency without unwanted off-target penalties, such as growth and yield reductions.

Although the inclusion of mass spectral imaging within plant metabolite research is rapidly increasing, significant technological and physical restraints remain a key limitation to widespread use. These limitations were recently reviewed by de Souza et al. (2020), where the authors discuss the technical challenges of spatial metabolomics, including the limited instrumental sensitivity that makes detection and/or quantification of metabolites that are present in minimal quantities difficult as well as the nature of the vast chemical variability of the metabolome, with no technique capable of visualizing every molecule within an organism [14]. Additionally, de Souza et al. touch on multi-cell- and single-cell-type approaches using mass spectrometry and MSI, discussing the broader implications and future directions of single-cell metabolomics, including integration with functional genomics and multiomics at the single-cell level.

### 1.2. Bulk Tissue Collections Dilute the Metabolic Phenotype

**Figure 1:**
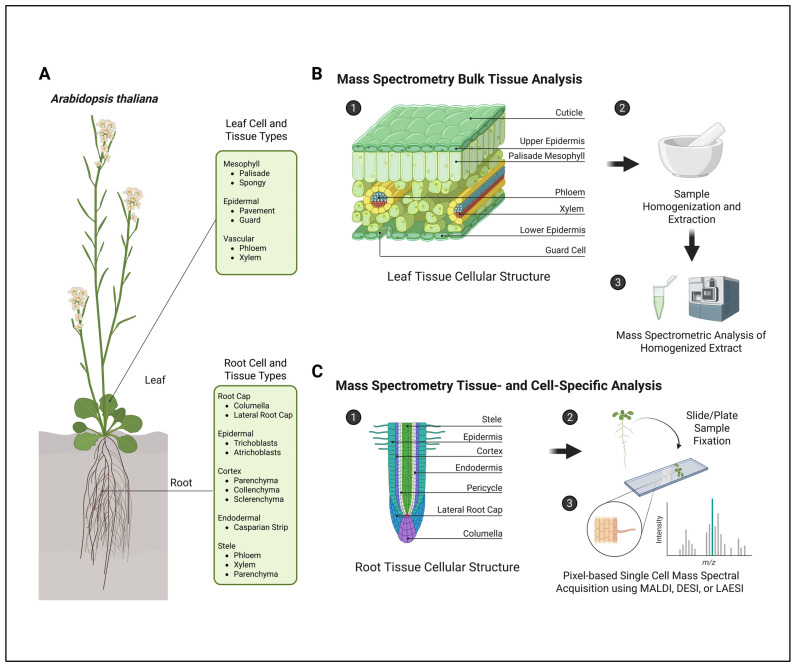
Comparison of mass spectrometry analyses performed using bulk tissue sample preparation versus spatial metabolomics workflows. (**A**) Individual tissue and cell types found within leaves and roots of *Arabidopsis thaliana*. (**B**) Schematic diagram of the leaf cellular structure and generalized sample preparation of leaf tissue samples for mass spectrometry bulk-tissue-type analyses. (**C**) Schematic diagram of the root cellular organization and generalized sample preparation for tissue-specific and single-cell-type spatial metabolomics analyses. Figure created in BioRender. Myers, R. (2025) https://BioRender.com/5lqoxwf.

Metabolomics research can be distinguished by the method of sample collection employed within the study (Figure 1). Historically, most metabolomics analyses have pooled various cell types and tissues together for homogenization into a single sample prior to metabolite extraction. As a result, metabolites cannot be mapped back to specific organelles, cells, or other locations within the tissue [19]. This not only hinders data analysis but also limits the conclusions that can be drawn from a given experiment, making it difficult to link metabolic changes to a specific tissue function. Additionally, metabolites that may play a substantial role within cell-specific responses can be challenging to detect and investigate since the metabolite is diluted within an extract from a pool of many heterogenous cells. However, bulk sample collection has still provided valuable insight into metabolic pathways, as it is the collection technique through which most information on plant primary and secondary metabolism has been elucidated.

Mass spectral imaging technologies can reduce or eliminate the need for bulk tissue analyses, as sample preparation is typically performed on a sectioned tissue for DESI-MSI and MALDI-MSI [20]. Additionally, MSI can also be combined with other technologies to enhance the study of bulk tissues. Proton nuclear magnetic resonance has been used by Honeker and colleagues (1H-NMR) in combination with MALDI-MSI to investigate the metabolic dynamics of drought responses in roots, with bulk metabolite analysis being performed through 1H-NMR and the labeled carbon flux being mapped through MALDI-MSI [21].

### 1.3. Current Perspectives and Potential Outlook

Published studies regarding instrumentational setups, data analysis techniques, and unique application studies incorporating MSI into metabolomic analysis have been increasing. As many as 378 articles are currently being published on MSI on a yearly basis. This number is expected to continue rising as MSI technologies become more widely adopted and instrument sensitivity and resolution continue to increase [22]. MSI and its respective technologies within plants have recently been the subject of multiple reviews [23,24]. Here, we describe recent advancements in technological development and publicly available software that can be utilized for MSI workflows. Additionally, we briefly describe the most recent advancements within emerging spatial imaging technologies, including laser ablation electrospray ionization (LAESI)-MSI and 3D NMR imaging. Finally, we conclude with an in-depth analysis of the most recent applications of DESI-MSI and MALDI-MSI within plant biology, with an emphasis on their use to study stress responses and specialized cell types.

## 2. Mass Spectral Imaging (MSI) Technologies

**Figure 2:**
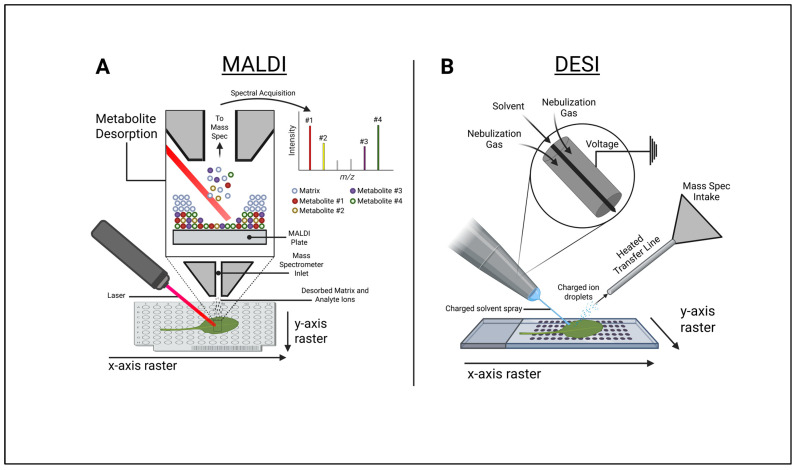
Illustrative comparison between matrix-assisted laser desorption ionization (MALDI) and desorption electrospray ionization (DESI) mass spectral imaging technologies. (**A**) Schematic diagram of typical MALDI mass spectral imaging ion generation and spectral acquisition of spatial data. (**B**) Schematic diagram of typical DESI mass spectral imaging ion generation and mass spectral intake. Figure created in BioRender. Myers, R. (2025) https://BioRender.com/y4pnozn.

Mass spectral imaging (MSI) technologies have rapidly improved since their original introduction. The most common techniques for MSI include matrix-assisted laser desorption ionization (MALDI) and desorption electrospray ionization (DESI) (Figure 2). These techniques are utilized in conjunction with mass spectral imaging software capable of mapping acquired mass spectral data back to individual pixels within a captured image. Other, newer techniques, including laser ablation electrospray ionization (LAESI) and spatial metabolite imaging techniques with nuclear magnetic resonance (NMR), have also emerged to reduce sample preparation and improve image quality. Here, we describe recent innovations in these technologies.

### 2.1. Matrix-Assisted Laser Desorption Ionization–Mass Spectrometry Imaging (MALDI-MSI)

MALDI-MSI is the oldest and most widely utilized metabolite imaging technology. MALDI technology was introduced to desorb ions from organic solids and later adapted for MSI by the Caprioli lab [11,25]. MALDI-MSI works by embedding a sample within a matrix coating on a conductive material surface. Following sample preparation, the embedded sample is subjected to a series of laser pulses, resulting in desorption and ionization of metabolites from the surface that can then be transferred to a mass analyzer. A series of mass spectra for the entire sample can be acquired while rastering laser pulses across a tissue section at controlled distances along the X and Y dimensions, resulting in a mass spectrum for each pixel. Historically, the pixel size was ~100–150 μm, but it is currently approaching 5 μm or less [26]. Increasing the resolution through technological development reduces the pixel sizes, making the MSI data of subcellular structures simpler to interpret, but also directly reduces the sensitivity as the number of metabolites within the pixel also decreases. In addition, increasing the resolution increases the data acquisition time and file size, as more pixels are needed to cover the same spatial distances [27]. Data acquisition times have improved with the development of faster lasers (i.e., 20 Hz in older MALDI systems compared to 2000 Hz or more in newer instruments). MALDI is often coupled with time-of-flight mass spectrometry but can be incorporated with other mass analyzers. Typical MALDI-MSI workflows utilize either atmospheric or vacuum conditions during ionization.

Innovations in MALDI systems are instrumental in advancing metabolomics in plant research and are the subjects of multiple papers. Recently, advancements in MALDI-TOF technology for plant tissue analysis were reviewed by Susniak et al. [28]. Susniak et al. explained that sample preparation and matrix application, with a focus on techniques like cryosectioning and lyophilization, are regarded as essential innovations for the preservation of metabolites during analysis. Additionally, sublimation and automated spraying systems were described as newer techniques that improve the spatial resolution of MALDI systems by increasing the purity and uniformity of the applied matrix [28]. Instrumental improvements, such as improved lasers, can allow for more accurate localization of analytes in samples. An attempt was recently made by Kompauer et al. to modify a commercially available AP-MALDI system with a newer laser focusing objective, resulting in a decrease in the laser ablation diameter from 4–6 µm to 1.4 µm, increasing the lateral resolution of the instrument to 1.4 µm [26].

The matrix coating of the sample surface influences the gas-phase chemistry and which metabolites are more readily desorbed and ionized by MALDI. Multiple groups have found that preparation of samples with a combination of different matrices allows for an increase in the number of detected metabolites [26,29,30]. Feenstra et al. performed an analysis of six different matrices to characterize which metabolites could be better detected within each matrix in maize kernels [26]. The authors showed that 1,5-diaminonaphthalene (DAN) could be used to detect acidic phospholipids and small molecules and that 9-aminoacridine (9AA) demonstrated effective detection of nucleotide-like compounds. Silver (Ag) allowed for detection of small molecules and phosphonoacetaldehyde. Additionally, 2,5-dihydroxybenzoic acid (DHB) was found to be an efficient matrix for large molecules such as proteins, lipids, and oligosaccharides. Iron oxide (Fe_3_O_4_) and tungsten oxide (WO_3_) matrices showed specificity for small sugars and triacylglycerols. A separate group had a similar approach for detecting metabolites within aerial tissue of grapevine leaves using the matrices DHB, α-cyano-4-hydroxycinnamic acid (CHCA), and 2,4,6-trihydroxyacetophenone (THAP) [30].

Increasing the spatial resolution has been an important area of research for the application of MALDI. Hansen and Lee successfully achieved five-micron, high-spatial resolution MS imaging; previously, ~fifty-micron resolution was used [31]. Additionally, Hansen and Lee aimed to develop a “multiplex MSI” data acquisition method to allow more data to be obtained on a single tissue in a single instrument run. They achieved this using four-micron laser spot size imaging of maize root cross sections with MALDI-MSI. It is anticipated that 3-micron MS imaging could be possible if further optics modifications were made to obtain a 2–2.5-micron laser spot size [31].

Recently, Yin et al. suggested that by introducing post-ionization methods and recycling waste molecules, the MSI resolution and efficiency can be increased and higher resolution images can be achieved [23]. Quantitative MSI can be achieved using stable labeled isotopes as an internal standard to quantify the metabolite imaged to provide an estimation of the concentrations and better describe the activity of metabolic pathways. The authors also suggested the use of whole plant imprinting for a more detailed view of complex metabolic processes as potential improvements for MSI workflows.

Sample preparation and preservation are other key issues in the field of spatial metabolomics. Preservation of sample tissues prior to and during spatial metabolic analysis is integral to generating high-quality MSI data. Electromagnetic-field-assisted frozen tissue planarization (EMFAFTP) is a novel technique discussed by Hu et al. and designed to be used in conjunction with MALDI to image plants [32]. This technique applies adjustable electromagnetic fields to gently press and flatten frozen plant tissue, reducing the matrix effects caused by tissue heterogeneity and allowing for enhanced and non-destructive metabolite identification from MALDI while keeping the spatial integrity intact.

### 2.2. Desorption Electrospray Ionization (DESI)

Electrospray ionization (ESI) is a common method for molecular ionization that is used to generate the ions necessary for mass spectrometry analyses. The technique was first employed for the analysis of biomolecules in 1984 and transformed the field by making analysis of large and complex molecular species possible [33,34]. One of the advantages of the technique is that it allows the analysis of biomolecules from solution, including nonvolatile biomolecules. This was a significant advancement as chemical ionization and electron ionization could previously only analyze volatile molecules. Since its inception, many groups have continued to modify and advance ESI technology. Recently, Takáts et al. found that electrospray ionization directed toward a surface can ionize and desorb analytes from that surface without sample preparation using ultrafine charged droplets that can form a thin liquid film [12]. They termed this modified technique desorption electrospray ionization (DESI). According to Takáts et al., analytes present on the surface of a sample are extracted into the liquid film, and the film becomes disrupted by continued spraying, allowing secondary droplets to become airborne. These droplets undergo “re-electrospray” ionization, producing gas-phase ions that can be directed into an intake line for mass spectrometry analysis. Additionally, DESI experiments can be fine-tuned by modifying the solvent composition to adjust the conductivity and affinity of the solvent, gas pressure, and sprayer angle/distance. These adjustments enable analyte-specific changes to improve the extraction efficiency. Morato et al. described porous polytetrafluoroethylene (PTFE) as extremely hydrophobic and often used as an analyte imprintation material within DESI experiments [35]. DESI has some advantages over MALDI, as it does not require a matrix to be used during sample preparation and allows for live sample analyses [36].

DESI technology continues to advance and is now commonly paired with mass spectrometry imaging (DESI-MSI) to visualize various metabolites in situ. Similarly to MALDI-MSI, mass spectra can be spatially collected and mapped back to the analyzed tissue through the process of rastering along the X and Y dimensions using the DESI sprayer as an ionization source [37]. The desorbed and ionized metabolites then enter a heated transfer line and can be measured using different types of mass analyzers, including time-of-flight, triple quadrupole, or orbitrap analyzers. The resultant mass spectra can then be mapped back to origin on the tissue sample on a per-pixel basis. Although DESI-MSI has advantages over MALDI-MSI, such as simpler sample preparation and straightforward analysis under ambient conditions, it also has important limitations to consider. Unlike MALDI-MSI, DESI-MSI is primarily a surface scanning technique that has limited depth of sample penetration for metabolic analyses. Additionally, DESI-MSI generally has lower sensitivity and reduced spatial resolutions (20–50 microns) as compared to MALDI-MSI [15]. However, many of these limitations can be accounted for by performing additional sample preparation steps and method modifications, such as performing mass spectral analysis on cryosectioned tissues and changing the chemical properties of the solvent spray [15].

The use of DESI-MSI within plant systems is rapidly increasing even though MALDI-MSI has previously been used much more frequently than DESI-MSI. One example of this emerging technology was demonstrated by Zhang et al., where the desorption spot created by DESI was used to raster scan plant root tissue and characterize the location of small molecules involved in plant metabolism, such as multiple tricarboxylic acid cycle metabolites, to determine the function and regulation within this metabolic pathway [38]. Attempts to combine DESI-MSI with other techniques to tailor the technology to experimental needs within plant systems have also been increasing. Recently, Wu et al. utilized a PTFE membrane to imprint metabolites from sage, gingko, and tea leaves [39]. Wu et al. showed that post-photoionization increased the sensitivity of DESI, allowing for greater coverage of compounds than DESI alone. This mapping of phenylpropanoid metabolic pathways in tea leaves provided valuable insights into the defense responses and structural pathways of these plants.

### 2.3. Laser Ablation Electrospray Ionization (LAESI)

Laser ablation electrospray ionization mass spectrometry imaging (LAESI-MSI) is a useful technology for mapping the metabolite distributions of untreated biological samples in vitro. In an LAESI source, a 2940 nm IR laser light is used to irradiate samples [40]. The irradiated material is quickly heated by the laser energy and vaporization of surface water molecules occurs. After the energy deposition induced by the laser becomes greater than the energy consumed in vaporization, the water molecules reach a superheated state. As the water returns to a relaxation state, energy is released and results in the ablation of surrounding metabolites. As most of these ablated metabolites consist of neutral particles, an electrospray ionization module is used to ionize the ablated material to enable analysis by a mass analyzer [40]. LAESI-MSI has the advantages of involving minimal sample separation and preservation of the natural environment in the tissue.

Most studies involving MSI within plants have utilized MALDI-MSI and DESI-MSI; however, applications using LAESI-MSI have been increasing in recent years. Li et al. showed that coupling ion mobility spectrometry (IMS) with LAESI-MSI on *Podophyllum peltatum* leaves can make it possible to differentiate compounds that would otherwise overlap [41]. They found that some metabolites, like diosmetin derivatives, were excluded from leaf veins, while others, like quercetin, were evenly distributed. Li et al. also indicated that IMS helped resolve four isobaric compounds at *m*/*z* 1566.22, which was not possible via conventional mass spectrometry.

LAESI-MSI can be used to analyze multiple types of plant tissues from different species. Kulkarni et al. analyzed the root metabolomes between native and range-expanding species [42]. This group performed LAESI-MSI imaging on the roots of seedlings to determine spatially resolved metabolic fingerprints and compare the overall concentration of metabolites from each plant. For the Centaurea plant genus, Kulkarni et al. found that there were 314 shared metabolites between the species and 49 unique metabolites for the range-expanding plant. However, for Geranium plants, the range expanding plant had four unique metabolites. It was indicated that mass spectral images of Centaurea plants had unique spatial regulation of metabolites, whereas metabolites found within the Geranium plants were similar in terms of the spatial distribution.

Through specialized sample preparation, LAESI-MSI technology has the capacity to explore the metabolome of single cell types within plant tissues. *Allium cepa* and *Fittonia argyroneura* were recently investigated for the metabolic responses at single-cell resolution by Taylor et al. [43]. In this study, LAESI was performed on cells extracted from *Allium cepa* epidermal tissues and a region of the *F. argyroneura* leaf. Using this type of cell collection process, Taylor et al. identified malic acid, mercaptoacetic acid, and maleic acid in *Allium cepa*. Malate and trisaccharide concentrations varied across *Alium cepa* cells. In *F. argyroneura*, catechol, furoic acid, and phthalide were localized in the vein region.

Modifications to LAESI-MSI have been investigated to increase the potential of the technology for ambient metabolite analysis. Laser ablation atmospheric pressure photoionization (LAAPPI) is an alternative form of LAESI that has been used to investigate the spatial distribution of phytochemicals in *Salvia officinalis* leaves [44]. LAAPPI-MSI can be used to directly profile plant metabolites, particularly terpenes and triterpenoids, without requiring sample preparation. The technique involves using a laser to ablate a thin layer of plant tissue, which is then ionized by atmospheric pressure photoionization, with the resulting ions detected by mass spectrometry. Atmospheric pressure photoionization (APPI) works by using ultraviolet light to excite molecules, causing them to ionize. This technique appears especially useful for ionizing non-polar molecules. This highlights a key advantage of LAAPPI-MSI in visualizing non-polar compounds, which can be difficult using other ionization methods.

### 2.4. Magnetic Resonance Imaging (MRI)

NMR and MRI technologies have been used as a gold standard to structurally characterize natural products from plants. NMR uses chemical shifts associated with 1H, 13C, 15N, and 19F nuclei to elucidate connectivity and absolute chemical structures. NMR-based MRI technologies are non-destructive and have been used to convert magnetic resonance properties to visualize the spatial distribution of compounds. However, MRI has rarely been used for plant metabolite imaging. Recent advancements in pulse programs, increased magnet strength, and data analysis tools have improved the resolution of NMR data; however, the spatial resolution remains a weakness of NMR/MRI applications relative to MS methods in spatial metabolomics. Chemical exchange saturation transfer (CEST) experiments have been shown to have a resolution of 50 μm and chemical shift imaging (CSI) experiments have been shown to have 300 μm resolution, which are significantly lower resolutions than MS methods [45].

Unlike mass spectrometry (MS), which suffers from ionization bias and requires reference standards, NMR allows for non-destructive and quantitative analysis of metabolites [46]. Traditionally, NMR is limited by low sensitivity and complex spectra. However, advances, including high-field magnets, cryoprobes, and ultrafast acquisition, have improved NMR performance. Multidimensional techniques like J-resolved, diffusion-ordered spectroscopy (DOSY), heteronuclear single quantum correlation (HSQC), and heteronuclear multiple bond correlation (HMBC) can typically resolve overlapping signals. These techniques can also reveal structural details without needing separation steps. Although DOSY and other techniques are still developing in plant studies, they show promise. This is more apparent when combined with tools like homonuclear correlation spectroscopy (COSY), total correlation spectroscopy (TOCSY), and high-resolution magic angle spinning (HR-MAS) NMR. However, making full use of NMR in plant metabolomics still depends on analytical innovation. Moving forward, advances in sensitivity, computation, and spectral libraries will be key to unlocking the full potential of NMR in plant metabolomics.

Recently, Mayer et al. explored a new method of using magnetic resonance imaging (MRI) to study the spatial and temporal distribution of plant metabolites in vivo [45]. Usage of CEST allows for non-invasive spatial metabolite acquisition by measuring the ability of metabolites to exchange protons with water molecules. MRI techniques may provide higher sensitivity than MS methods for compounds that exhibit ion suppression, such as highly polar compounds like sugars and zwitterionic compounds like amino acids. Alongside displaying the technological advancement, Mayer et al. demonstrated the utility and versatility of this technology by applying CEST to measure the spatial and temporal shifts in sugar and amino acid accumulation within barley pods, kiwi fruit, sugar beet, sugarcane stem, and potato [45]. However, this technique cannot differentiate between individual sugars and amino acids, significantly limiting its ability to identify specific metabolites. Even with its current limitations, advancements in this technology are promising for the development of new methods for NMR/MRI-based spatial metabolic identification in the future.

### 2.5. Mass Spectral Imaging Software

A current challenge in MSI is the processing and analysis of the enormous quantity of data collected. During an MSI experiment, each pixel that is collected contains its own associated mass spectra, making data analysis significantly more complicated than typical mass spectrometry experiments. Therefore, developing software capable of understanding and visualizing the cumulative data is vital. Most MSI instruments have their own dedicated analysis software, such as the SciLS and MetaboScape from Bruker and the Waters HDI 1.7 from Waters Corporation. However, commercial software may have limitations, and a wide range of open-source packages are publicly available online. These have varied user interfaces, adaptability and often require more computational knowledge on the part of the researcher. This section highlights the current efforts toward developing accessible open-source MSI analysis tools.

Cardinal v.3 is one of the most popular open-source R/Bioconductor-based mass spectrometry imaging analysis packages that can analyze various biomolecules, such as metabolites, lipids, and peptides, with data obtained using MALDI and DESI [47,48]. The software is tailored to handle large-scale imaging datasets and supports visualization of ion intensity maps. It can handle data preprocessing, including baseline correction and normalization, as well as more advanced tasks, including feature selection, unsupervised clustering, and spatially aware classification [47]. Statistical analyses incorporated into Cardinal include principal component analysis (PCA), spatial shrunken centroids (SSCs), and k-means clustering, which allow users to identify spatially resolved metabolic patterns directly from raw or centroid data. An important feature of Cardinal is its capability of processing large datasets quickly on a personal computer, increasing the accessibility for most users. The software is in its third iteration and requires previous background knowledge of using the R programming language.

Metabolite Imager is an additional open-source Java-based program for analyzing MSI data, developed using the freeware Netbeans v7.XIDE software [49]. It allows researchers to evaluate metabolite distributions and explore variations within tissues at the granular level. To generate a 2D image within Metabolite Imager, the X and Y coordinates of specific metabolite positions are determined by a rectangular raster scan, allowing for determination of metabolite localization. The program also allows researchers to define specific parameters based on the target metabolites and ions, expected adducts (*m*/*z*), and image dimensions. Metabolite Imager incorporates algorithms to visualize co-localization of metabolites, further aiding in the study of precursor–product relationships in situ, as well as customized and accurate image processing.

Multi-MSI Processer (MMP) is one of the most recently developed open-source platforms designed to streamline the MSI data analysis process. Released in 2023, it accepts common types of MSI data files (.raw, .mzXML, and .imzML), which makes it compatible with AFADESI, MALDI, SIMS, and DESI [50]. It can read raw data and create 3D data with *m*/*z* alignment, ROI selection, background ion removal, and sample quality control using PCA and thresholding.

The inclusion of artificial intelligence (AI) within spatial metabolomics data analysis processing is an emerging topic and will likely play a prominent role in MSI software in the future [19]. As AI development continues to accelerate and be incorporated into mass spectrometric analysis, it will be interesting to see how rapidly AI assistance is incorporated by the mass spectrometry community and how it may improve data analysis pipelines. Collaborative platforms for sharing MSI data, such as METASPACE, use machine learning to facilitate metabolite identification [19].

## 3. Applications of Spatially Resolved Metabolomics in Plant Biochemistry

**Figure 3:**
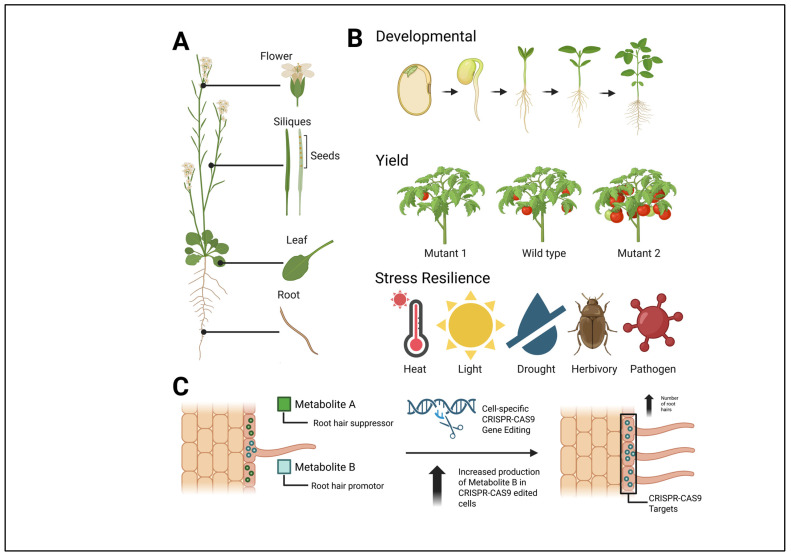
Potential applications of mass spectral imaging technologies in plants. (**A**) Types of plant tissue that can be analyzed for tissue-specific or single-cell-specific mass spectral data analyses. (**B**) Aspects of plant physiology that can be investigated using mass spectral imaging approaches, including developmental processes, yield traits, and stress resilience mechanisms. (**C**) Theoretical approach to changing the anatomy of root tissue through the application of CRISPR-CAS9 gene editing and targeted metabolite modifications. Figure created in BioRender. Myers, R. (2025) https://BioRender.com/nxrkrck.

Mass spectral imaging incorporates several emerging techniques to spatially resolve metabolites from various organisms for analysis. Here, we describe recent applications of MSI within plants. Metabolic analysis using MSI can be combined with other types of analyses, such as microscopy, transcriptomics, and physiological assays, to yield novel information that would not be possible to obtain without the acquisition of spatial information. The acquired data is analyzed for potential pathway discovery and to further our understanding of tissue and metabolite function. This provides novel insights into specific, diverse roles of primary and specialized/secondary metabolites in cell growth and development, defense, stress response, immunity, and cell signaling. As the body of research continues to grow, new applications of spatial metabolomics will lead to advancements in areas such as abiotic and biotic stress resistance, plant growth and development, and overall crop yield (Figure 3).

### 3.1. Mass Spectral Imaging of Root Metabolites

Root systems are critical to plant health, being responsible for numerous vital activities, including water acquisition, plant stabilization, and nutrient uptake [51]. Plant metabolites play essential roles in these processes, both within the root and as root exudates to the rhizosphere [52,53]. Spatial metabolomics enables characterization of metabolite function based on co-localization within a sample. This helps elucidate previously unknown or understudied relationships between metabolites and metabolic pathways.

#### 3.1.1. MSI of Root Metabolites in Abiotic Stress Responses

Abiotic stress plays an important role in crop yields globally, reducing yields by more than 60% on average when compared to record yields [54]. Research on abiotic stress responses can be leveraged for the development of more robust and stress-resilient crops, which are vital to securing the sustainability of the food supply over the coming century as the environment becomes increasingly erratic and detrimental to crop growth and yield [55]. The spatial metabolome responses to abiotic stress represent an emerging area of plant stress biology research that has the potential to deliver novel insights into abiotic stress tolerance and identify potential metabolic avenues for improving stress resiliency. Following confirmation of the severity of stress impacts through typical stress markers, such as the measurement of reactive oxygen species levels and detection of programmed cell death, MSI can be used to investigate the mechanisms through which plants attempt to coordinate metabolic responses during stress events. Recent research has investigated spatial metabolism under different types of abiotic stress, including phosphorus (P) deficiency, aluminum (Al) toxicity, salinity, drought, and cold stress in the roots of different species such as *Arabidopsis thaliana*, *Marchantia polymorpha* (common liverwort), *Avicennia marina* (mangroves), *Phoenix dactylifera* (date palms), and *Asparagus officinalis* (asparagus) [17,56,57].

#### 3.1.2. MSI of Root Metabolites in Abiotic Stress Responses—Nutrient Deficiency and Toxicity

The nutrient balance and deficiency play a defining role in the regulation of plant growth and yield under abiotic stress conditions. Nutrient deficiencies alone are enough to significantly impact crop yields, and nutrient deficiencies can make plants more vulnerable to other types of stress, such as drought, heat, and disease [58,59,60]. Recent spatial metabolomics studies have incorporated workflows to investigate nutrient responses within the root. Gomez-Zepeda et al. utilized MALDI-MSI high-resolution tandem mass spectrometry (HRMS/MS) for organic acid (OA) mapping in *Arabidopsis thaliana* and *Marchantia polymorpha* roots and root exudates under phosphorus deficiency and aluminum toxicity conditions [56]. They found that OAs were differentially regulated in roots under stress conditions by both P deficiency and Al toxicity, while malate and other OA exudates increased under stress conditions. Interestingly, Gomez-Zepeda et al. showed that the spatial localization of root exudates was not heavily affected by stress, but the internal root citrate localization was dependent on stress. Additionally, phosphorus-deficient conditions resulted in citrate accumulation within the meristematic zone and root cap, while in P-sufficient conditions, citrate was found in the apex and root hair, indicating that citrate localization may be stress dependent [56].

#### 3.1.3. MSI of Root Metabolites in Abiotic Stress Responses—Drought and Salinity

Salinity and drought stress are also of particular interest within the abiotic stress research community. High salinity currently impacts as much as 20% of irrigated land throughout the world, causing between 20% and 50% reductions in the overall crop yield [60]. Similarly, drought stress can result in yield reductions ranging between 15% and 60%, with reductions as high as 90% being seen during extreme drought events [61,62]. With both salinity and drought stress projected to impact larger segments of agricultural land over the coming decades, improving crop resiliency against these stresses is vital. Recent work by Oyarce et al. applied MALDI-MSI to analyze the distribution of root metabolites in mangrove (*Avicennia marina*) and date palm (*Phoenix dactylifera*) roots, finding that mangrove roots accumulated increased metabolites involved in lignin and suberin production while date palm roots accumulated increased metabolites with roles in amino acid biosynthesis [17]. Additionally, the authors found that amino acid osmoprotectants accumulated in date palm roots while carbohydrate osmoprotectants accumulated in mangrove roots. Their results indicated that plants may exhibit differentially unique, spatially resolved regulation of metabolites to respond to drought and salinity stress, making future studies of spatial metabolic regulation in response to salinity and drought promising avenues for discovery of stress resilience mechanisms.

#### 3.1.4. MSI of Root Metabolites in Abiotic Stress Responses—Cold

Additional spatial metabolomics root studies have been performed that analyzed asparagus roots at the outer, middle, and inside root layers by MALDI-MSI. Witzel and Matros found decreased fructan accumulation in asparagus roots when plants were transferred from cold storage to greenhouse conditions [57]. The decrease in fructans indicates a potential protective role of fructans during cold stress in roots.

#### 3.1.5. MSI of Root Metabolites in Metabolic Mapping

Although understanding the metabolic responses of plants during stress is currently of key importance to the plant research community, many additional root metabolites that have roles in other aspects of plant development and physiology have been identified and are currently of interest for investigation using spatial metabolic analyses. Numerous metabolites with specific and unique roles are found within roots. By understanding the distribution of these metabolites, we can better understand the processes involved in their biosynthesis, metabolic regulation, and function. Additionally, multiple plant species contain metabolites with medicinal properties within the root that may be of interest to both the plant and human health research communities. Many of these metabolites have recently been investigated at a spatial level within tissue specific root layers.

Carbohydrates are a key class of metabolic molecules that play vital roles in the regulation of multiple biochemical processes within plant tissues. In addition to being required for energy metabolism and used as structural carbon precursors for many compounds, they also have roles as signaling molecules and regulators of plant–microbe interactions [63]. Recent applications of MSI have investigated the spatial distribution of carbohydrates and related downstream products. The spatial distribution of root carbohydrates and gallotannins was investigated by Li et al. in *Paeonia lactiflora* and *Paeonia suffruticosa* to elucidate the pathways of gallotannin biosynthesis through MALDI-MSI [64]. They found that various monosaccharides and polysaccharides in *Paeonia suffruticosa* were found to localize in the cortex and phloem, while in *Paeonia lactiflora*, they were found in the cortex and xylem. Additionally, they discovered that the gallotannin 1,2,3,4,6-penta-O-galloyl-β-D-glucopyranose (5GG) was found in plant roots but did not show any specific pattern of spatial distribution. Interestingly, the oxidative products of 5GG were localized in the phloem of *P. suffruticosa* and the xylem and cortex of *P. lactiflora*, indicating potential systematic movement of the metabolites from their location of synthesis to specific distant tissues. These findings built upon prior findings derived from atmospheric pressure–scanning microprobe matrix-assisted laser desorption ionization–mass spectrometry imaging (AP-SMALDI-MSI) on *P. lactiflora* to spatially resolve metabolites, where the locations of gallotannins, monoterpene glucosides (MGs), and various primary metabolites, such as amino acids and oligosaccharides, were spatially determined [65]. In their previous work, multiple gallotannins, including pentagalloylglucose to nonagalloylglucose, were predominantly localized in cork and xylem, whereas monoterpene glucosides showed broader distributions across multiple tissue types [65]. Additionally, MALDI-MSI has been used in plant root metabolic mapping in other plant species. In 2020, Li et al. mapped the distribution of 18 bioactive constituents in *Salvia miltiorrhiza* (red sage), showing that phenolic acid and tanshinones were concentrated in the roots, with tanshinones only located in the root periderm [66].

MALDI-MSI has been applied most frequently for spatial metabolic analysis within plant roots; however, DESI-MSI-based approaches to mapping the spatial distribution of metabolites in roots have been increasing in popularity. In Wang et al., *Curcuma longa* (turmeric) roots were recently analyzed by DESI-MSI to visualize the distribution of 130 different metabolites, including amino acids, curcuminoids, flavonoids, and terpenoids [67]. They found that multiple metabolites have distinct spatial distributions, including β-elemene localized in the rhizome, cortex and epidermis of roots, and curcumene and curcolone localized in the root cortex and xylem. Additionally, the spatial distribution of monoterpenoid indole alkaloids (MIAs) in various tissue types of *Rauvolfia tetraphylla* (wild snakeroot) by DESI-MSI was recently investigated by Kumara et al., with the roots being found to be the primary site of MIA biosynthesis [68]. As alkaloids have known roles in plant defense and may have medicinal benefits, MIAs may play a role within root stress responses.

Similarly, Du et al. analyzed the spatial distribution of 52 saponins in the tea plant *Camellia sinensis* by DESI-MSI [69]. Saponins are used in the food industry and have pharmaceutical potential. Du et al. found that the root epidermis and cortex were found to contain many di- and trisaccharide saponins [69].

Alternative technological approaches to DESI-MSI have been recently used to enhance root metabolic studies. Air-flow-assisted desorption electrospray ionization–mass spectrometry (AFADESI-MSI) has been applied by Guo et al. to explore the interspecies metabolomics of *Pueraria lobata* (PL) and *Pueraria thomsonii* (PT) [70]. PL and PT are medicinally relevant plants with different therapeutic effects; however, the reason behind their varying medicinal properties is not clear. Through AFADESI-MSI, which utilizes increased air flow speeds relative to typical DESI experimental workflows to improve the ionization efficiency and sensitivity of large molecules, Guo et al. found that key isoflavones like genestin, daedzin, and puerarin are up to 52× more abundant in PL roots than in PT roots. Additionally, PT had elevated sugars, starch, and cellulose in its roots. It is possible that this variation in the content of isoflavones and other metabolites may play a role within the determination of the medicinal properties of each species.

Ultimately, these studies help to lay the groundwork for future explorations of metabolic pathways and provide potential avenues for metabolic engineering in plants. While there are many plant metabolites with perceived medicinal roles, an understanding of the spatial localization of medicinal plant metabolites can be leveraged to develop plants with greater concentrations of targeted metabolites for industrial use [71].

### 3.2. Mass Spectral Imaging of Aerial Tissue

In addition to root studies, spatial metabolomics has been utilized in studying plant aerial tissues. Upon the sensation of stress, both rapid and long-term changes in the composition of the metabolome occur within aerial tissues, impacting the plant defense, signaling, development, and stress response capabilities of the plant [72,73,74]. Understanding how this tissue responds at both the rapid and long-term timescales is of key importance for development of stress-resilient crop species.

#### 3.2.1. MSI of Aerial Metabolites in Abiotic Stress Response—Drought and Water Deficit

Aerial responses to abiotic stresses are expected to lead to novel insights useful in mitigating crop losses and improving crop efficiency. Recently, MALDI-MSI was applied by Balasubramanian et al. to spatially resolve metabolites in distinct cell types undergoing water deficit stress [75]. They found that palisade mesophyll cells had an increased abundance of specific flavonoids and fatty acids during water deficit stress. Alternatively, they showed that vascular cells showed reduced fatty acids and increased sugars under water deficit. This study suggests that stress-related metabolites relevant to water deficit stress are regulated in a cell-type-specific manner within aerial tissue and that different cell types have different regulatory roles in stress resiliency.

Abiotic stress can also have major effects on non-leaf aerial tissues, such as fruits. In Asakura et al., MALDI-TOF-MSI was used to image green (mature but not ripe) and red (mature and ripe) tomato fruits [76]. The authors found differential metabolite accumulation and co-localization of inositol, β-alanine, and fatty acids, including palmitic acid, stearic acid, and oleic acid, between fruit pericarp, mesocarp, and seeds under control and drought conditions [76]. Further, Lemaire-Chamley et al. showed that there is a correlation between the metabolite localization and the different stages of ripening using spatialized NMR and metabolic analysis to assess the different tissues of a tomato plant [77]. They analyzed five distinct tomato fruit tissues (exocarp, mesocarp, columella, placenta, and locular tissue surrounding the seedlings) using NMR alongside enzymatic starch assays and liquid chromatography with diode array detection (LC-DAD), allowing for the analysis of different metabolites and isoprenoids at varying developmental stages. Lemaire-Chamley and coauthors also indicated that the metabolic signatures in different tissues were found to be present in each of the individual tissues and to be correlated between differing developmental stages. Interestingly, their results showed that exocarp contained γ-tocopherol as a central metabolite and an essential antioxidant in reducing oxidative damage [77]. Additionally, lycopene and β-carotene increased greatly during ripening, especially in internal tissues.

#### 3.2.2. MSI of Aerial Metabolites in Leaf and Fruit Wounding Responses

Mechanical wounding in plants can be caused by a variety of factors, including pathogens, pests, drought, and cold, which result in negative impacts such as growth reductions and, in severe cases, plant death [78,79]. To mitigate this damage, plants have developed sophisticated responses to wounding, including rapid systemic signaling, hormone modulation, and defensive metabolite accumulations [80,81,82,83]. Studying these responses can yield novel insights into developing insect- and pathogen-resistant crops.

Zhang et al. recently mapped the spatial arrangement of various phytohormones, including their precursors, in wounded leaves compared to unwounded leaves with DESI-MSI [84]. They showed that wounded leaf regions accumulated significantly more jasmonic acid (JA), salicylic acid (SA), indole-3-acetic acid (IAA), and abscisic acid (ABA) than unwounded controls. Specifically, JA was found to have the most intensity directly within the wounded region, while SA, IAA, and ABA appeared more broadly within the wounded leaf, reiterating that JA has a key role in activation of wounding responses at the local site of injury [84].

Similarly, Veličković et al. focused on determining the lipids involved in the wounding stress response in tomato leaves. This study spatially resolved specific lipids with MALDI-FTICR-MSI and MALDI-TimsTOF-MSI at three time points (before wounding, 30 min post-wounding, and 60 min post-wounding) with three different leaf sections (apex, middle, base). They showed that the lipidome responded to wounding stress within 30 min but recovered to normal levels within 60 min [85]. Veličković et al. also indicated that wounded leaves accumulated alpha-linolenic acid and/or linoleic acid, phosphatidylcholines, phosphatidylethanolamines, and triacylglycerols, while sulfoquinovosyldiacylglycerols, diacylglycerols, and phosphatidic acids declined, demonstrating the role of these lipids in lipid signaling during wound response.

Leaf wounding was also recently explored in leaves of the tea plant *Camellia sinensis* to determine the metabolic changes caused by insect herbivory. By mimicking the feeding mechanism of tea green leafhoppers on leaves, Dai et al. showed that increased abundance of catechins, quercetin, and quercetin glycosides in the leaf vein compared to unwounded leaves occurred, indicating a role of these compounds in the herbivory response specifically within the vasculature [86].

Understanding the effects of wounding stress on fruits is also critical to decrease crop loss post-harvest. Nakamura et al. investigated wounding by harvesting green and red tomato fruits, inducing wounding, and subsequently incubating the fruits for one week in non-sterile conditions. They then used MALDI-MSI to visualize over 30 metabolite-related ion species across distinct anatomical fruit regions, including the pericarp, locular tissue, and vascular bundles, discovering that α-tomatine, a rapid-response defense metabolite, was highly localized in wounded tissue near the vasculature [87]. Additionally, they found that the metabolite esculeoside A was found to be localized in unwounded and ripening tissues. The data implies that while ripening occurs in a tomato, it has increased esculeoside A to protect against insect damage and impart anti-inflammatory effects.

#### 3.2.3. MSI of Aerial Metabolites in Metabolic Mapping

Plant aerial tissues are primary sites of many distinct metabolic processes. These tissues exhibit distinct metabolic compartmentalization that can be determined with MSI. Studies can provide insights into species-specific traits that shape the spatial organization of both primary and specialized metabolites. The following studies demonstrate how MSI has been applied to aerial tissues to determine metabolite localization, infer biosynthetic pathways, and guide applications ranging from crop improvement to medicinal plant development.

Many studies have used high-resolution MSI to localize metabolites within aerial tissues at sub-tissue levels. Korte et al. mapped maize leaf metabolites using MALDI–linear ion trap–Orbitrap at a 10 µm resolution, which revealed localization of flavonoids in the epidermis and sugars in the vascular bundles [88]. These findings provided a framework to further link transcriptomics to gene expression. Similarly, Nakabayashi et al. mapped the sulfur-containing metabolite asparaptine A and its precursor arginine in green asparagus using matrix-assisted laser desorption ionization–Fourier transform ion cyclotron resonance–mass spectrometry (MALDI-FTICR-IMS). Buildup of these compounds was found around developing lateral shoot tissues, suggesting that this may be the site of asparaptine A biosynthesis and that it may have a role in shoot development and plant architectural determination [89].

Medicinal plants have been of special interest in terms of metabolic mapping of aerial tissues. Multiple studies have recently been performed that investigated the spatial localization of metabolites within various types of medicinal plant leaves. Li et al. used MALDI-MSI to investigate the epidermis, vascular bundle, and cortex of red sage, assessing the differential distribution of salvianolic acids and tanshinones, which have pharmaceutical relevance [66]. Similarly, Du et al. investigated the localization of a different pharmaceutically relevant type of compound (saponins) using DESI-MSI. They found large amounts of cinnamoyl tetrasaccharide saponins within tea plants, tea buds, and leaves [69]. Additionally, Liu et al. performed metabolic profiling in *Dendrobium noblie* to visualize the spatial distribution and describe a synthetic pathway for dendrobine by identifying 34 metabolites with UHPLC-QToF-MS and MALDI-ToF-MSI [90]. These reports show that spatial metabolomics may be particularly useful for identifying ways in which pharmaceutically relevant metabolites can be produced at a larger scale through the enhancement of tissue-specific production pathways.

MSI is also suited to studying dynamic changes in the metabolite distribution across developmental stages in aerial tissues. Wu et al. recently investigated *Gelsemium elegans* using DESI-MSI to uncover shifts in the alkaloid distribution over different stages of life, finding that the diversity of alkaloids decreased and shifted in position from mesophyll to leaf vein from seedling to maturity [91].

### 3.3. Mass Spectral Imaging of Specialized Cell Types

Plants have numerous specialized cell types, including leaf, trichome, stomatal guard cells, seed, seed coat, and root border cells, among others. These specialized cells have unique compartmentalized metabolic pathways that regulate cell-type-specific metabolic processes. Analyzing these specialized metabolites provides a more comprehensive understanding of the mechanisms and processes underlying cell-specific functions. However, traditional metabolomics approaches often use bulk tissue analysis, which can be limiting (as described earlier in Section 1.2). Recent MSI applications have investigated specialized cell types to provide greater understanding of the functions of these cells and how metabolites influence their function.

#### 3.3.1. Trichomes

Trichomes are variable, specialized cells involved in multiple, well-documented roles in plant defense [92]. While trichrome structures vary between plant species, *Arabidopsis thaliana*, a standard model, has unicellular trichomes [93]. To explore the metabolic profile of trichomes in *Arabidopsis*, Hieta et al. utilized laser ablation atmospheric pressure photoionization–mass spectrometry (LAAPPI-MS) to spatially resolve metabolites in the trichomes of leaf tissue at a single-cell resolution [94]. This study found a significantly greater abundance of fatty acids and their respective esters in trichomes, as opposed to other leaf regions. Additionally, the trichome base contained highly accumulated flavanol glycosides, which may offer surface protection for plants against UV radiation.

#### 3.3.2. Stomatal Guard Cells

Guard cells are required for stomatal opening and closing, gas exchange regulation, and transpiration. Characterizing the metabolites within guard cells can potentially guide approaches to improve the stomatal dynamics during growth and stress responses [95]. Dong et al. recently employed MALDI-ToF-MSI to analyze the concentration and spatial distribution of malate and other carbohydrates in stomatal guard cells, and to evaluate the roles of the acetate–malate shunt in drought resistance and osmoregulation [96]. In *Arabidopsis thaliana*, they found that reduced accumulation of acetate–malate shunt acetyl-CoA synthetases resulted in guard cells accumulating reduced malate and diminished stomatal opening.

#### 3.3.3. Seed and Seed Coat

Seeds, a critical organ for plant reproduction, provide a controlled environment for next-generation plant germination [97]. Improvement of the seed durability, robustness, and nutritional composition are of specific importance to the plant science community [98]. Multiple research groups have recently begun investigating the metabolic composition of seeds using MSI.

Enomoto et al. recently investigated seeds from the common bean, *Phaselous vulgaris* L., using a combination of DESI-MSI and LC-ESI-MS to map various oxylipins implicated in seed development [99]. One notable metabolite that they identified and measured was jasmonic acid (JA), which is an important plant hormone that is heavily involved in plant responses to biotic and abiotic stress, such as cold, drought, and herbivory damage [100]. They discovered that JA was found to be primarily localized in the seed coat and radicle, indicating that its potential roles within the regulation of seed embryogenesis and dormancy may be due to modulation of this region [99].

#### 3.3.4. Seed and Seed Coat—Drought and Salinity

Additionally, the effects of drought and salinity stresses are of particular importance in seeds because both factors influence the seed yield and germination [101,102]. Liu et al. recently utilized both LC-MS/MS metabolic profiling and MALDI-MSI spatial metabolomics within cotton wildtype and drought-resistant cultivar seeds [103]. Their study reported that untargeted metabolomics identified 17 metabolites of interest within the drought-resistant seeds, many of which were implicated in water retention, osmotic regulation, and reactive oxygen species (ROS) scavenging. Additionally, amino acid metabolism, wax and flavanols were differentially accumulated within the drought-resistant cultivar seeds. Interesting, when differential lipid accumulation was analyzed via MALDI-MSI, they found that multiple lipids were found to be differentially accumulated in either the entire seed or solely within the cotyledon in response to salt and drought stress.

#### 3.3.5. Seed and Seed Coat—Pathogen Response

Additional studies have investigated other aspects of stress, including the effects of pathogen-induced stress responses on seed metabolite accumulation. Righetti et al. recently employed atmospheric pressure (AP)-SMALDI-MSI, a modified version of MALDI-MSI, to determine differences in the defense metabolite accumulation and localization in wheat (*Triticum aestivum* and *Triticum durum*) kernels infected by *Fusarium* spp. [104]. They found that coumaroylagmatine was localized in the pericarp outer cuticle in infected seeds, suggesting a potential role in antifungal activity and cell wall strengthening. Additionally, they showed that diacylglycerols accumulated exclusively in the pericarp of infected seeds, while galactolipids accumulated only in the endosperm of healthy seeds.

#### 3.3.6. Root Border Cells and Nodules

Root border cells are an interest to the Sumner lab and important contributors to root biology, with potential functions within root development, rhizosphere regulation, and stress responses. However, their functions remain poorly understood [105]. Watson et al. recently integrated metabolomics and transcriptomics to understand how *Medicago truncatula* root border cells differ from the root tips using GC-MS and LC-MS analyses [106]. They discovered that high levels of flavonoids, such as 7,4′-dihydroxyflavone, and hormones, including jasmonic acid and salicylic acid, were found to accumulate within border cells compared to the adjacent root rip. These compounds have known roles in plant pathogen defense and cell signaling, indicating a specific role of border cells in protecting the root tip and supporting positive plant–microbe interactions.

Understanding nodule development is an additional area of particular interest within root biology as the nodule serves as the primary environment for symbiotic nitrogen-fixing bacteria within legume crop species [107]. In situ spatially resolved metabolic profiles of soybean root nodules infected with different strains of *Bradyrhizobium japonicum* were recently investigated by Agtuca et al. using LAESI-MS analysis [108]. They induced soybean nodules through either wildtype *B. japonicum* inoculation or through infection with *B. japonicum* mutants, including strains that lacked nitrogenase activity (*nifH* mutant) and strains with altered lipid metabolism (*sacpd-c* mutant). In nodules induced by the WT strain, they showed that the infection zone contained metabolites involved in nitrogen fixation, including glycerophospholipids, purines, and gibberellins. Additionally, in nodules induced by a *nifH* mutant, there was increased jasmonic acid, indicating a potential stress response due to nitrogen deficiency. Interestingly, in the *sacpd-c* mutant, they found increased soyasaponins and organic acids, and there were spatial differences in the accumulation of these, with soyasaponins being localized only in the outer cortex, while heme B and purines were enriched in the infection zone.

#### 3.3.7. Metabolic Transport and Intracellular Communication

Both primary and secondary metabolites have been shown to be biosynthesized in specific tissues or organs within a plant and to undergo long-distance transportation to other distant tissues [109,110]. The “source-to-sink” relationship is the most described metabolic transport phenomenon, with plant leaves being known to produce an excess of sugars that are then transported to tissues of the plant requiring higher levels of sugars for growth, such as developing roots and shoots [111]. Transport of metabolites is primarily mediated through phloem and xylem cells of the plant vasculature, but there have been reports of long-distance transport within non-vasculature plants [110,111,112]. Additionally, rapid plant responses to stress have been shown to be mediated through various wave-like signals that propagate from a local site of stress sensation to distant tissues [83,113,114,115,116,117]. These signals can produce stress-specific changes in metabolite accumulation that allow for acclimation and protection against the stress that was sensed within the environment [73,118]. However, most studies that have investigated the transport of metabolites or the accumulation of metabolites in response to molecular signaling events during stress have analyzed the metabolome through bulk tissue analyses. Further studies are needed to investigate the signal-induced metabolic changes or movement of metabolites during development at a cell-specific level with MSI, as this is expected to provide additional insight into cell-specific functions during the plant-wide coordination of development and stress responses.

### 3.4. Integration of Mass Spectral Imaging and Omics Technologies

By pairing spatial metabolomics data with other omics technologies, such as proteomics, transcriptomics, genomics, and epigenomics, researchers gain deeper insights regarding metabolic processes. Prior to the adoption of MSI technologies, traditional combinatorial omics studies were performed on individual, sorted cell types to gather cell-type-specific data. For example, Brechenmacher et al. aimed to characterize the proteome of soybean (*Glycine max*) root hairs [119]. Brechenmacher et al. utilized total protein analyses paired with HPLC-MS/MS to identify peptides and generate an accurate mass and time (AMT) protein database of soybean root hairs. Similarly, Watson et al. combined metabolomics and transcriptomics to investigate the molecular differences between root border cells and the root tip in *Medicago truncatula* [106]. These combined approaches better defined the roles of border cells in plant pathogen defense and cell signaling.

More recently, numerous publications have combined spatial metabolomics with other spatial omics approaches. Ge et al. utilized a multiomics approach, including RNA-sequencing and airflow-assisted desorption electrospray ionization–mass spectrometry (AFADESI-MSI), on cotton somatic embryos to identify metabolites and genetic markers in distinct spatial regions and determine the relationship between the transcriptome and the metabolome [120]. Interestingly, they discovered that L-arginine was localized within cotyledons, while α-linoleic acid was localized in the cortex. Of the gene markers detected, AATP1 and DOX2 were of interest due to their significant transcriptional abundance during embryogenesis. Ultimately, they concluded that AATP1 and DOX2 act as negative regulators in embryo formation with this combined spatial omics approach. DOX2 is known to metabolize a-linoleic acid, perhaps explaining the negative regulation as reduced fatty acid biosynthesis leads to embryonic developmental defects.

Additionally, Jozwiak et al. used MALDI-MSI to localize glycoalkaloids that are produced by the GAME15 enzyme pathway in *Solanum nigrum* [121]. By using spatialized aspects of MSI, they were able to co-localize metabolites and their associated enzymes. Their orthogonal data from the MALDI-MSI and RNA sequencing analyses indicated that GAME15 enzymes are a co-localized group of enzymes that synthesize glycoalkaloids. Additional spatially resolved metabolomics and RNA sequencing by Jozwiak et al. showed that this localized enzyme assembly complex regulates the production of steroidal alkaloids and saponins.

## 4. Concluding Remarks

Mass spectral imaging, and its incorporation into experimental workflows, is a powerful technology that has the potential to revolutionize the field of plant metabolomics. Recent applications within multiple areas of plant science have shown proof of concept that these types of analyses can and do lead to novel insights regarding the functions of previously characterized metabolites through the association with the specific tissue of origin. With combinations of multiple environmental stressors becoming increasingly prominent and severe throughout the world, solutions are needed to mitigate the effects of these stressors within crop plants and avoid potential disaster in relation to the global food supply [122]. Rapid adoption of MSI is crucial to achieving this goal as it can potentially accelerate novel findings regarding stress-related metabolites and enhance the depth of our understanding of metabolic pathways, allowing for new genetic engineering approaches to improve plant stress tolerance. Additionally, developing a deeper understanding of the locational aspects of metabolite function is expected to enable novel approaches to improving plant development and yield during both stress and stress-free conditions. Identification of tissue- or cell-specific metabolite accumulations that have roles during development or under stress conditions has the potential to allow selective single-cell-type genetic engineering of these pathways, which will enhance stress tolerance or yield without resulting in negative off-target metabolic effects. Potential synchronization and merging of this technology with other omics methods, such as single-cell RNA sequencing, is also particularly promising for generating a large amount of integrated transcriptomic and metabolic data in tandem.

Overall, MSI within plant science is extremely promising and may fundamentally change our approach to plant metabolomics in the future. As the technology continues to improve, both MALDI- and DESI-MSI have the potential to be widely utilized for single-cell-level metabolite mapping. Currently, MALDI-MSI is likely preferable in most plant single-cell studies, as it is capable of higher resolution (5 µm) compared to the ~20 µm resolution range that can be achieved through a fully optimized DESI source [15,26]. MALDI-MSI can be the only available option for mapping metabolites in some plant cells, such as meristematic cells, due to their small at 5 µm [123]. However, as the DESI-MSI resolution continues to increase, it may ultimately become better suited for studying stress responses within plants as it has the potential to ambiently analyze metabolites from living tissues. Advancements in DESI technology are needed before this can occur, as most currently available DESI imaging platforms are designed to analyze samples that have been attached to a slide, fixed, or cryosectioned before analysis [20]. Additionally, the limited penetration of the DESI sprayer into the plant tissue can be problematic, as acquiring data on non-surface-level tissues may require additional sectioning of samples along the z-axis. Ultimately, as the technology matures, the choice of which of these to employ may largely be determined by instrument accessibility.

## Data Availability

No new data were created or analyzed in this study. Data sharing is not applicable to this article.

## Abbreviations

ABA	Abscisic Acid
AFADESI-MSI	Air-Flow-Assisted Desorption Electrospray Ionization–Mass Spectrometry
Ag	Silver
Al	Aluminum
AMT	Accurate Mass and Time
AP-MALDI	Atmospheric Pressure–Matrix-Assisted Laser Desorption Ionization
APPI	Atmospheric Pressure Photoionization
AP-SMALDI-MSI	Atmospheric Pressure–Scanning Microprobe Matrix-Assisted Laser Desorption Ionization–Mass Spectrometry Imaging
CEST	Chemical Exchange Saturation Transfer
CHCA	Cyano-4-HydroxyCinnamic Acid
COSY	Homonclear COrrelation SpectroscopY
CSI	Chemical Shift Imaging
DAN	1,5-DiAminoNaphthalene
DESI	Desorption Electrospray Ionization
DHB	2,5-DihyHroxyBenzoic acid
DOSY	Diffusion-Ordered SpectroscopY
EMFAFTP	ElectroMagnetic-Field-Assisted Frozen Tissue Planarization
ESI	ElectroSpray Ionization
Fe_3_O_4_	Iron Oxide
FTICR-IMS	Fourier Transform Ion Cyclotron Resonance–Mass Spectrometry
GC	Gas Chromatography
HMBC	Heteronuclear Multiple Quantum Coherence
HRMS/MS	High-Resolution Tandem Mass Spectrometry
HSQC	Heteronuclear Single Quantum Coherence
HR-MAS	High-Resolution Magic Angle Spinning
IAA	Indole-3-Acetic Acid
IMS	Ion Mobility Spectrometry
LAAPPI	Laser Ablation Atmospheric Pressure Photoionization
LAESI	Laser Ablation Electrospray Ionization
LC-DAD	Liquid Chromatography–Diode Array Detection
JA	Jasmonic Acid
MALDI	Matrix-Assisted Laser Desorption Ionization
MGs	Monoterpene Glucosides
MIAs	Monoterpenoid Indole Alkaloids
MMP	Multi-MSI Processer
MRI	Magnetic Resonance Imaging
MSI	Mass Spectral Imaging
NMR	Nuclear Magnetic Resonance
OA	Organic Acid
P	Phosphorus
PCA	Principal Component Analysis
PTFE	Porous PolyTetraFluoroEthylene
QtoF	Quadropole Time of Flight
ROI	Region of Interest
ROS	Reactive Oxygen Species
SA	Salicylic Acid
SIMS	Secondary Ion Mass Spectrometry
SSC	Spatial Shrunken Centroids
THAP	2,4,6-TriHydroxyAcetoPhenone
Tims-TOFMS	Trapped Ion Mobility Spectrometry–Time-of-Flight Mass Spectrometry
TOCSY	Total Correlation Spectroscopy
WO_3_	Tungsten Oxide
5GG	1,2,3,4,6-penta-O-Galloyl-β-D-Glucopyranose
P-AA	9-AminoAcidine
